# Editorial

**DOI:** 10.1120/jacmp.v7i1.2248

**Published:** 2006-02-21

I want to welcome a couple of new Associate Editors to the JACMP. Salahuddin Ahmad joins as Associate Editor in Radiation Oncology Physics, while Geoffrey Clarke joins as Associate Editor in Medical Imaging. Salahuddin and Geoffrey, thanks for being a part of the JACMP and welcome to the Board of Editors. All of our Editors volunteer their services; it is only with such generous contributions that we are able to bring the JACMP to you every quarter without cost.

I also want to mention our publishing partner, Multimed, Inc. The American College of Medical Physics, sponsor of the JACMP, has appreciated the high quality and cost effectiveness of the web publishing services provided by Multimed. The ACMP is expanding its relationship with Multimed to include web hosting services, registration for our annual meeting, dues payments and etc. We expect the ACMP to better meet the professional needs of the Medical Physics community through its relationship with Multimed, Inc. So on behalf of both the ACMP and JACMP, we appreciate Multimed as our media partner as we reach and serve the worldwide Medical Physics community.

Michael D. Mills, PhD

**Table:** 

The Journal of Applied Clinical Medical Physics	
**Editorial Board 2006**	
Editor‐in‐Chief	Michael Mills
Deputy Editor‐in‐Chief	Timothy Solberg
Associate Editor‐at‐Large	Richard Stark
**Associate Editors**	
Radiation Oncology Physics	Nzhde Agazaryan, Salahuddin Ahmad
	B. Gino Fallone, John Gibbons,
	Michael Herman, Janelle Molloy,
	Matthew Podgorsak, Nikos Papanikolaou,
	Mehrdad Sarfaraz, Lu Wang
Medical Imaging	Geoffrey Clarke, William Pavlicek
Radiation Measurements	Larry DeWerd
Radiation Protection & Regulations	James Deye
Nonionizing Topics	Bhudatt Paliwal
Other Topics	Timothy Solberg
Book/Media Reviews	James Smathers
Editorials	John Horton
**Journal Production Team**	
Proofreader	Heather Shand
Copy Editor	Betty Robinson
Layout Editor	Laura Shand
Manuscript Manager	Patricia Walker

